# An Open-Label, Noncomparative, Multicenter Study to Evaluate Efficacy and Safety of NASHA/Dx Gel as a Bulking Agent for the Treatment of Fecal Incontinence

**DOI:** 10.1155/2010/467136

**Published:** 2010-12-27

**Authors:** Giuseppe Dodi, Johannes Jongen, Fernando de la Portilla, Manoj Raval, Donato F. Altomare, Paul-Antoine Lehur

**Affiliations:** ^1^Università degli Studi di Padova Hospital Clinica Chirurgica, Via Giustiniani 2, 35128 Padova, Italy; ^2^Proktologische Praxis Kiel, Beselerallee 67, 24105 Kiel, Germany; ^3^Coloproctology Section, Department of Surgery, Virgen del Rocio University Hospital, Avenida Manuel Siurot s/n, 41013 .Seville, Spain; ^4^St. Paul's Hospital, C313-1081 Burrard Street, Vancouver, BC, Canada V6Z 1Y6; ^5^Coloproctological Unit of Bari Policinico di Bari Piazza, University of Bari, Giulio Cesare 11, 70124 Bari, Italy; ^6^Clinique Chirurgicale, chu-Hôtel-Dieu, Institut des Maladies de l'Appareil Digestif, 1 Place Alexis Ricordeau, 44093 Nantes Cedex, France

## Abstract

Fecal incontinence (FI) is the involuntary loss of rectal contents through the anal canal. Reports of its prevalence vary from 1–21%. Studies, have demonstrated a positive effect on FI symptoms with injectable bulking agents. This study evaluated the safety and efficacy of NASHA/Dx gel in the treatment of FI. One hundred fifteen eligible patients suffering from FI received 4 injections of 1 mL NASHA/Dx gel. Primary efficacy was based on data from 86 patients that completed the study. This study demonstrated a ≥50% reduction from baseline in the number of FI episodes in 57.1% of patients at 6 months, and 64.0% at 12 months. Significant improvements (*P* < .001) were also noted in total number of both solid and loose FI episodes, FI free days, CCFIS, and FIQL scores in all 4 domains. The majority of the treatment related AEs (94.9%) were mild or moderate intensity, and (98.7%) of AEs resolved spontaneously, or following treatment, without sequelae. Results of this study indicate NASHA/Dx gel was efficacious in the treatment of FI. Treatment effect was significant both in reduction of number of FI episodes and disease specific quality of life at 6 months and lasted up to 12 months after treatment.

## 1. Introduction

Fecal incontinence (FI) is the involuntary loss of rectal contents through the anal canal [1]. Reports of its prevalence vary from approximately 1% to 21% [2–6]. Fecal incontinence affects up to 8% of the adult population over the age of 65 years [5–7]. In the elderly population both sexes are equally affected, but in younger populations, (aged 25–45) FI is 8 times more common in women than in men [6, 8, 9]. 

Obstetric trauma is recognized as a common cause. Patients may present with FI in the postpartum period or sometimes with an onset delay of several years. Trauma during delivery may cause incontinence by direct rupture of the anal sphincters or by overstretching of the pudendal nerves. Fecal incontinence can also be caused by colorectal disease, trauma, neurological disorders, or congenital abnormalities [10–12]. 

The severity of FI may vary over time as the condition is influenced by external factors such as physical exercise, stress, concurrent illness, and diet. Fecal incontinence can be a source of embarrassment for those affected and has a great negative impact on the quality of life [13]. 

Treatment regimens usually begin with dietary and lifestyle modifications and pharmacotherapy such as fiber therapy and antidiarrheals. Overlapping sphincteroplasty is the most common surgical therapy for FI. This surgery works for isolated sphincter injury cases but is not indicated in patients without sphincter injuries or in patients with multiple sphincter defects in different locations. Moreover, recent studies have demonstrated that the efficacy of sphincteroplasty may diminish over time [14].

 New surgical alternatives to overlapping sphincteroplasty such as sacral nerve stimulation (SNS) [15–17], Secca's procedure [18–20], artificial bowel sphincter (ABS) [21–23], and dynamic graciloplasty [24–26] have been developed and are increasingly used in the treatment of FI [27]. 

Nonreactive bulking agents have been used successfully in aesthetics, vesicoureteral reflux (VUR) in children, and stress urinary incontinence (SUI) for many years [28–30]. As a result, noninvasive treatment of FI with bulking agents has developed [31]. Small studies have demonstrated a positive effect on FI symptoms with a low rate of complications with this method of treatment [32–36]. 

NASHA/Dx gel consists of nonanimal stabilized hyaluronic acid and dextranomer microspheres (Solesta, Oceana Therapeutics, Edison, NJ). It is biocompatible, nonallergenic, and showing no sign of distant migration of the dextranomer [37]. The material in NASHA/Dx is identical to Deflux, which has been safely used for the treatment of children suffering from VUR (i.e., the retrograde flow of urine from the bladder to the ureter [38–40]) over the past 10 years [41–43]. Also, this identical material is approved for SUI treatment in the European Union and Canada under the name Zuidex; it is not approved in the United States (US) for SUI. Following transanal submucosal injection of NASHA/Dx, the dextranomer facilitates the ingrowth of fibroblasts and collagen between the microspheres as hyaluronic acid is degraded. The bolus is thus consolidated with endogenous tissue, stabilizing its volume for a sustained durable response.

The purpose of this study was to evaluate the efficacy and safety of NASHA/Dx gel as an injectable bulking agent in the treatment of FI. Safety was evaluated for a total of 12 months after the last treatment.

## 2. Methods

This was an open-label, Noncomparative, one group, prepostdesign, 15-center study performed in Europe and Canada. A total of 115 eligible patients (100 female, 15 male) with a mean age of ~62 years (range 30–80 years) suffering from FI were treated with NASHA/Dx gel (Table 1). Informed consent was obtained from patients prior to initiation of any study-related activities. The study was conducted in accordance with the World Medical Association Recommendations Guiding Physicians in Biomedical Research Involving Human Patients amended by the 52nd World Medical Association (WMA) General Assembly, Edinburgh, Scotland 2000 [44], and the notes of clarification on paragraphs 29 and 30 dated 2002 and 2004, respectively [45–47]. The protocol was approved by the Ethics Committee of each hospital involved in the study. Collection of data, organization and construction of the database, and all statistical analyses and outputs were performed and retained by the sponsor. The statistician involved in the analysis is an employee of the sponsor. All authors had access to the database and clinical study report and assume responsibility for the completeness and accuracy of the content of the paper. Approximately two thirds of the patients had been symptomatic for <5 years, and the most common underlying cause of the FI was attributed to obstetric injuries (32% of patients) closely followed by neurogenic cause (30%). Patients were screened for baseline data and eligibility at a screening visit up to 12 weeks before the first treatment visit.

## 3. Inclusion Criteria

At screening, the investigator interviewed the patient regarding the existence of FI defined as the inability to control loose or solid stool as well as the severity of FI, to obtain the baseline CCFIS. Eligible patients were adults of ages 18–80 suffering from FI (i.e., a Cleveland Clinic Florida Incontinence Score (CCFIS) ≥5 at baseline and ≥4 FI episodes over a 28-day period as recorded in a patient diary). The duration of FI prior to inclusion had to be ≥12 months, and the patients must have failed conservative treatment.

## 4. Exclusion Criteria

Patients were excluded from the study if they were not determined to suffer from FI and had anal or rectal malformation, prolapse, fissures, rectal varices, stenosis, implants, or complete external sphincter disruption. Likewise, patients with a history of anorectal surgery within the last 12 months, or previous stapled transanal rectal resection (STARR) or stapled hemorrhoidectomy <2 cm above the dentate line, were excluded. Patients with histories of anorectal tumors, malignancies, or chemotherapy within the last 6 months prior to the study, or previous radiation therapy with signs of radiation injury in the area to be treated, were excluded from the study.

Patients with a medical history of human immunodeficiency virus (HIV) infection or any condition with severe compromised immune defense, or on immunosuppressive therapy, bleeding diathesis or on anticoagulant therapy (such as warfarin, heparin, or heparin-like substance), anorectal sepsis, anorectal bleeding, and active Inflammatory Bowel Disease (IBD), and women that were pregnant or breastfeeding, of childbearing potential not practicing adequate contraception or planning to stop such contraception within the first year of the study or 6 months after partum, were also excluded from participation in the study.

## 5. Study Design

Eligible patients received 4 injections of 1 mL NASHA/Dx gel. Endoanal ultrasound was performed as a part of the physical exam at screening to assess the intactness of the sphincter. Bowel evacuation by fleet enema was compulsory prior to treatment. The use of local anesthetic was optional, and the need for prophylactic antibiotics was determined by the investigator.

The injections were performed through an anoscope, and the NASHA/Dx gel was placed in the deep submucosal layer of the anal canal approximately 5–10 mm above the dentate line at the anorectal junction. Injection was done at ~30° angle, and the needle was kept in place for 15–30 seconds, to prevent premature leakage of the solution over the puncture opening.

One month after the first treatment visit, the patients were offered retreatment of up to 4 × 1 mL NASHA/Dx gel, provided that the criteria for retreatment were fulfilled. To be eligible for retreatment, the patient had to remain incontinent according to the patient's FI diary; have no persistent adverse effect; have no other medical reason against retreatment; be willing to receive retreatment. The optional retreatment was meant to enable individual adjustment of the dose. If the patient was continent according to patient's diary and had improved to his/her satisfaction, no retreatment was to be performed.

The number of FI episodes per 24 hours was recorded in the diaries. The recordings were specified as leakage of at least 2 mL solid or loose stool. Gas or stainings were not to be recorded. The words “at least 2 mL” were repeated on each page of the diary so it was quite clear to the patients what should be recorded. Controlled bowel movements without accidents, urgency (need to hurry to the toilet) and use of bowel medication (antidiarrhea drugs, such as Imodium [loperamide] and Lomotil [diphenoxylate/atropine]), fiber products, and others were recorded. There were no recordings of gas or staining only. The number of incontinence-free days was also collected from the patient diaries. For a Diary to be considered valid, at least 21 days must have been completed with ≥3 FI episodes recorded, and the diary must have been filled in according to the instructions on the diary.

Primary efficacy was based on the assessment of FI episodes (loose or solid stool, not gas) and was measured by proportion of responders at 12 months after last treatment. A responder was defined as a patient with ≥50% decrease from baseline in the number of FI episodes (28-day patient diary data) of solid or loose stool. Additional efficacy evaluations at 6 and 12 months after last treatment included a change from baseline in the CCFIS [48, 49] and achange from baseline in Fecal Incontinence Quality of Life Scale (FIQL) [50] in countries where FIQL was available. Response in CCFIS was defined as having ≥30% reduction in CCFIS compared to baseline. The choice to use cut points of a 50% reduction from baseline in number of FI episodes and a >30% reduction in the CCFIS score, as thresholds, was to be consistent with that of other published reports and as the authors agreed that changes of this magnitude would represent a clinically meaningful change to the patients [36, 51]. 

The safety population was defined as all patients who were treated with study product. All analyses were performed on the safety population.

## 6. Safety

The safety of NASHA/Dx gel treatment was measured by the incidence, relationship, and severity of treatment emergent adverse events (AEs) reported during the study. Followup visits were performed at 1, 6, and 12 months after the last treatment. At followup visits, rigid proctoscopy (or flexible sigmoidoscopy) was performed, and patients were assessed for the presence or absence of AEs. Any changes in concomitant medications were recorded. The fecal incontinence Diary was collected, and a new diary was distributed.

## 7. Statistical Methods

The sample size of ~100 patients was not based on a statistical calculation. Using a sample size of 100 patients (~7 patients from each center), the maximum length of the 95% confidence interval (i.e., units) for the proportion of responders at 12 months was 20 percentage units (occurs at a proportion of 50% responders). By including 100 patients, there was approximately 99% probability to observe at least 1 event of an AE with a hypothetical prevalence of 4.5% and approximately 63% probability when the prevalence is 1%. Hence, 100 patients lead to a reasonable precision in the efficacy estimates and also facilitate findings of adverse events that are less common. The primary efficacy objective was to calculate the proportion of responders based on a change from baseline in the total number of incontinence episodes at 12 months, together with a 2-sided 95% confidence interval based on the normal approximation to the binomial distribution. The variable number of FI episodes had no upper limit, and therefore the Wilcoxon one-sample test was used to assess change from baseline together with a 2-sided 95% confidence interval based on the normal approximation to the binomial distribution. Continuous variables with a limit on minimum and maximum outcome (i.e., CCFIS, FIQL, incontinence-free days, number of days with antidiarrheal medication, and number of antidiarrheal medication doses) were analyzed using a onesample *t*-test (change from baseline) together with a 2-sided 95% confidence interval assuming normality. All tests were performed at the 5% significance level. *P*-values ≤.05 were considered statistically significant. No adjustment of *P*-values for multiple testing was performed.

## 8. Handling of Missing Data

Primary analyses were based on observed data. However, a last-observation-carried-forward (LOCF) technique was also used where the last performed efficacy assessment was carried forward to impute any subsequent missing values. Diary data for visit 3 (i.e., at 1 month) was however not used. If no efficacy assessments were available neither at the baseline nor at any followup visit, the assessment and the change from pretreatment value were missing in the analysis.

Partly missing data in the patient diary was handled as follows. If incontinence data were completed for ≥14 days, the number of FI episodes was scaled to number per 28 days in the following manner; (A) the number of FI episodes observed, divided by number of days with incontinence data, multiplied by 28. (B) The number of incontinence-free days was scaled in a similar manner. If <14 days of incontinence data were completed, the incontinence data at that time point was regarded as missing and handled according to the rules above.

Partially missing data in 1 of the 5 domains in CCFIS was left missing. Missing data for questions included in the FIQL was handled as follows. If ≥50% of the questions in 1 domain were completed, the mean value of the completed items replaced the missing responses. Consequently, if <50% of the questions in 1 domain were completed, the domain was regarded as missing and handled according to the rules above.

## 9. Results

**Figure 1:**
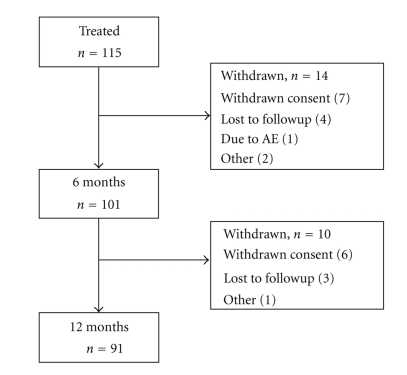
Disposition of patients.

Of the 115 patients treated with NASHA/Dx gel, a total of 14 patients withdrew or were lost to followup at 6 months (*n* = 101), and additional 10 patients withdrew or were lost to followup at 12 months (*n* = 91). Of the 24 patients that withdrew or were lost to followup, 13 withdrew consent, 7 were lost to followup, 3 withdrew for other reasons, and 1 patient withdrew due to an AE (see Figure 1). All enrolled patients (i.e., 115) received at least 1 treatment with NASHA/Dx gel, and 38 (33%) patients received an additional (2nd) treatment.

Protocol deviations were considered to be major if they would potentially influence the primary efficacy results (i.e., the evaluation of number of FI episodes and subsequent responder rates at 12 months). In total, 27 protocol deviations in 25 patients were classified as major. The 25 patients affected by the major protocol deviations were excluded from the per-protocol analysis of the primary objective at 12 months. Only patients with valid diaries at both baseline and at 12 months were included in the primary analysis. In total, 86 patients (i.e., 74.8% of the analyzed safety population) had evaluable diaries both at baseline and at the 12month visit and were consequently included in the final primary efficacy analysis. At baseline, the number of patients recording a valid patient diary was 114; this number was reduced to 100 patients at 6 months and 88 patients at 12 months. Since one patient had a missing diary at baseline, change from baseline could only be calculated for 99 and 87 patients at 6 and 12 months, respectively. Since one patient had a value of zero FI episodes at baseline, responder rates could only be calculated for 98 and 86 patients at 6 and 12 months, respectively. The patient recorded zero episodes at baseline but treated because they claimed having had more episodes in interview with the investigator. However, the diary, which was source data, was never changed. This incident was classified as major deviation, and the patient remained in the study for ethical reasons and safety followup.

At 6 months after last treatment, 56 out of 98 (57.1%) evaluable patients had ≥50% reduction from baseline in the number of FI episodes. At 12 months, the responder rate was 64.0% when based on observed cases in the analyzed safety population. The excluded patients comprised 5 patients with invalid FI diaries at baseline or at the 12-month visit and 24 patients who withdrew study participation prior to the 12-month visit. All efficacy data at 6 months are summarized in Table 2, and all efficacy data at 12 months are summarized in Table 3.

The reduction from baseline in number of FI episodes, recorded in the 28-day diary, was statistically significant at both 6 months (*P* < .001) and 12 months (*P* < .001) after last treatment. When based on observed cases in the analyzed safety population, the median change from baseline in number of FI episodes at 6 months was −7.0 (−58.9%) episodes (baseline 16.0). The median change from baseline in number of FI episodes at 12 months was −7.6 (−70.2%) episodes (baseline 15.0).

The increase in mean number of incontinence-free days was significantly higher at 6 and 12 months after treatment compared to baseline (*P* < .001 at both 6 and 12 months). The mean change from baseline was +6.9 and +7.1 incontinence-free days at 6 and 12 months after last treatment, respectively. The mean number of incontinence-free days was 20.9 at 6 months and 21.2 at 12 months after last treatment.

There was a statistically significant (*P* < .001) reduction from baseline in number of controlled bowel evacuations where the patients had to hurry to the toilet (as recorded in the 28-day diary) at 6 months (i.e., −6.1 instances); and the reduction from baseline was sustained at 12 months (*P* < .001) (i.e., −7.5 instances).

Improvement from baseline in Cleveland Clinic Florida Incontinence Score was statistically significant at both 6 months (*P* < .001) and 12 months (*P* < .001). In patients evaluable at both baseline and at 6 months (analyzed Safety Population, OC), the mean CCFIS was reduced from 13.5 at baseline to 9.2 at 6 months. Similarly, the mean CCFIS was reduced from 13.4 at baseline to 8.7 at 12 months.

The Fecal Incontinence Quality of Life (FIQL) questionnaire is divided into 4 domains: lifestyle, coping/behavior, depression/self perception, and Embarrassment. The lower the FIQL score, the lower quality of life. Statistically significant increases from baseline were observed at 6 and 12 months for all 4 FIQL domains. The results in the individual domains are summarized in Tables 2 and 3.

A total of 154 AEs were reported by 70 patients during the study. Of these events, 79 were assessed as related to the study treatment. The majority of the treatment-related AEs (94.9%) were assessed to be of mild or moderate intensity and most (92.4%) were classified as nonserious. Nearly all (98.7%) of AEs were resolved spontaneously, or following treatment, without sequelae. A total of 20 AEs, reported by 14 patients, were classified as serious. Six serious AEs (reported by 4 patients) that were deemed as possibly related to the study treatment comprised one case each of perineal abscess, rectovaginal septum abscess, and rectal abscess, as well as a case of concurrent rectal prolapse, proctalgia, and rectal hemorrhage. One patient died due to cardiac failure and was reported as a serious AE and determined to be unrelated to the study treatment. The most prevalent AEs (i.e., ≥3 events) are presented in Table 4.

Fever was fairly common after treatment. A total of 7% of patients reported pyrexia that were assessed by the investigator as related to treatment. All of these events commenced shortly after treatment and all resolved within a week of the onset. Two events required no treatment; 5 events were treated with antipyretics or anti-inflammatory agents and only 1 event with antibiotics.

A total of 6 cases of anorectal abscess were reported in the study. Three of these events commenced within the 1st week after treatment, 2 during the 3rd week after treatment, and 1 event after 130 days. All of these events resolved after treatment. In 3 cases, the abscess was incised and drained, followed by treatment with antibiotics. In the remaining 3 cases, no local surgical exploration was performed, and the patient was treated with antibiotics only. In the single instance where material was sent for microbiological cultures the results showed bacteroides fragilis. None of the patients developing abscess received prophylactic antibiotics prior NASHA/Dx injection.

## 10. Discussion

Currently, the injection of bulk-enhancing agents into the anal canal area for the treatment of FI is considered in its infancy [52]. Several different types of agents have been investigated (i.e., collagen, silicon beads, carbon beads, and NASHA/DX), and the results of these trials reveal low complication rates and a mild-to-moderate effect on incontinence symptom improvement [32–36]. 

Danielson and colleagues have reported on the use of NASHA Dx gel as an injectable anal canal implant for the treatment of FI [36]. The results of their study demonstrated a 50% reduction in the number of incontinence episodes compared with pretreatment values in 44% of treated patients at 6 months and 56% of treated patients at 12 months. In that study, no long-term side effects or serious adverse events were reported.

In the current study, the majority of patients were improved after treatment with NASHA/Dx gel. The proportion of patients having ≥50% reduction in number of FI episodes was 57.1% at 6 months after treatment and 64.0% at 12 months after treatment, indicating that the achieved effect is sustained for up to a year. This conclusion is supported by the observations in mean change from baseline in number of FI days were similar at 6 and 12 months after treatment; 59.2% and 65.1%, respectively. The treatment effect was also confirmed in analyses of the CCFIS. In addition to the frequency of FI episodes, this scale also includes other aspects of the disease such as lifestyle alteration and use of pads. The mean CCFIS was significantly lower at 6 and 12 months after treatment compared to baseline, and the reduction observed after 6 months remained at 12 months after treatment. Moreover, a similar pattern was observed for the patients' results in Fecal Incontinence Quality of Life instrument. All four domains measured by the FIQL were improved at 6 months after treatment, and the effect remained at 12 months after treatment. A number of the AEs related to treatment were indicative of perioperative infection which is not unexpected with treatments procedures in the anorectal region [53]. The results of safety and efficacy assessments noted in this trial are consistent with the results of a previous trial by Danielson et al. [36]. 

The NASHA/Dx gel (i.e., Solesta) used in this trial is the same substance marketed in the EU and Canada under the name Zuidex and is approved in those arenas for the treatment of SUI. In the US, this same substance (marketed under the name of Deflux) is approved for the treatment of VUR in children. It is not approved in the US for the treatment of SUI.

There have been several reports of the formation of sterile abscesses associated with the use of Zuidex in the treatment of women with SUI [54–56]. The formation of sterile abscesses in women with SUI following surgical intervention (tension-free vaginal tape, intervaginal slingplasty, etc.) is quite common [57–84]. However, in the pediatric population, the safety of NASHA/Dx gel (i.e., Deflux) for the treatment of VUR has been demonstrated to be quite good with very few reports of sterile abscess formation [29, 37, 85–87]. 

When considering the safety of NASHA/Dx gel for the treatment of FI, as is the case in this clinical trial and in a previous study by Danielson et al. [36], correlations between the safety profiles of Zuidex in the treatment of women with SUI, or Deflux in children for the treatment of VUR are difficult to make. This is due in part to the safety profiles associated with each product, the naturally high incidence of abscess formation in the treatment of women with SUI, the differences in patient populations and indications for usage, and the differences of application site. What is clear is the need for future long-term safety studies in the application of NASHA/Dx gel in the treatment of FI.

## 11. Limitations

Since the study did not include any reference arm it is not possible to determine the extent of placebo effect in the use of NASHA/Dx gel in the treatment of FI. Since no placebo arm was, included it is not possible to determine whether the observed proportions of related/unrelated AEs reflect underreporting of unrelated AEs. In addition, no valid stool consistency scale was used to better understand the exact nature of each patient's FI. In some cases, specific data points were missing, and the LOCF method was employed to impute missing data. Also validated pain scores and/or patient satisfaction scales may have added to the overall assessment of Solesta as a novel clinical option for FI patients.

## 12. Conclusions

The results of this study indicate that the use of NASHA/Dx gel was efficacious in the treatment of FI. The treatment effect was significant at 6 months and lasted up to 12 months after treatment, both in reduction of number of FI episodes and disease-specific quality of life. An additional advantage for the use of NASHA/Dx gel as a treatment for FI as opposed to treatment with injectable silicone biomaterial (PTQ) or Injectable synthetic calcium hydroxylapatite ceramic microspheres (Coaptite) is that it does not require the use of anesthesia.

## 13. Conflict of Interests

G. Dodi, M.D., has received support in the form of research grants from QMed AB, Uppsala, Sweden. He has not received honoraria for lectures or served as a consultant. He has no other financial disclosures to declare. J. Jongen, M.D. has received honoraria for lectures from Dr. Kade, Berlin, Germany, Ethicon Endosurgery, Norderstedt, Germany, Boehringer-Ingelheim, Ingelheim, Germany, Dr. Falk, Freiburg, Germany, and Eli Lilly & Co. there are no other financial disclosures. F. de la Portilla, Ph.D., M.D. has received honoraria for lectures from Ethicon Endosurgery, Medtronic SA, PALEX medical, MSD, Nycomed, Prostrakan Farmaceutica. He has served as a consultant for Ethicon Endosurgery, Medtronic SA, PALEX medical, MSD, Prostrakan Farmaceutica; and he has received support in the form of research grants from the Governments of Spain and Andalucía. F. de la Portilla, Ph.D., M.D. has received support in the form of research grants from Q-Med AB, Uppsala, Sweden. He has not received honoraria for lectures or served as a consultant. He has no other financial disclosures to declare. D. F. Altomare, M.D. has received support in the form of research grants from Q-Med AB, Uppsala, Sweden. He has not received honoraria for lectures or served as a consultant. He has no other financial disclosures to declare. P. A. Lehur, M.D., has received honoraria for lectures from Ethicon Endosurgery, Medtronic SA, American Medical Systems. He has served as a consultant for Ethicon Endosurgery, Medtronic SA, American Medical Systems, Coloplast, THD spa; and he has received support in the form of research grants from CEREC, University hospital of Nantes.

## Figures and Tables

**Table 1:** Patient demographics.

		(*n* = 115)
Female	*n* (%)	100 (87.0)
Male	*n* (%)	15 (13.0)
Age, years	Mean (range)	62.5 (30.5–80.3)
Body mass index, kg/m^2^	Mean (range)	26.2 (16.6–41.5)

*n*: number of patients; %: percent of study analyzed safety population.

**Table 2:** Changes from baseline at 6 months.

	Baseline	6 months	Change from baseline	*P*-value
Patients with ≥50% decrease from baseline in number of FI episodes, *n*(%), SE	0	56 (57.1) 0.050	56 (57.1) 0.050	N/A
Total number of FI episodes, mean (SE)	20.8 (1.71)	11.1 (1.84)	−9.7 (1.71)	<.001**
Total number of solid FI episodes, mean (SE)	9.5 (1.28)	5.7 (1.28)	−3.9 (1.32)	<.001**
Total number of loose FI episodes, mean (SE)	11.3 (1.21)	5.4 (0.78)	−5.8 (1.14)	<.001
Total number of FI-free days, mean (SE)	14.0 (0.73)	20.9 (0.73)	6.9 (0.73)	<.001*
Number of controlled bowel evacuations, mean (SE)	22.5 (2.07)	27.0 (2.31)	4.5 (1.86)	.015*
Cleveland Clinic Florida Incontinence Score, mean (SE)	13.5 (0.36)	9.2 (0.50)	−4.3 (0.47)	<.001*
Fecal Incontinence Quality of Life (FIQL) Scores				
Lifestyle, mean (SE)	2.4 (0.10)	3.0 (0.10)	0.5 (0.10)	<.001*
Coping/behavior, mean (SE)	1.8 (0.07)	2.3 (0.09)	0.6 (0.10)	<.001*
Depression/self perception, mean (SE)	2.6 (0.10)	3.1 (0.10)	0.5 (0.09)	<.001*
Embarrassment, mean (SE)	1.8 (0.08)	2.5 (0.10)	0.6 (0.10)	<.001*

SE: standard error.

*One-sample *t*-test; **Wilcoxon onesample test.

**Table 3:** Changes from baseline at 12 months.

	Baseline	12 months	Change from baseline	*P*-value*
Patients with ≥50% decrease from baseline in number of FI episodes, *n*(%), SE	0	55 (64.0) 0.052	55 (64.0) 0.052	N/A
Total number of FI episodes, mean (SE)	20.6 (1.90)	9.6 (1.81)	−11.0 (1.90)	<.001**
Total number of solid FI episodes, mean (SE)	9.6 (1.41)	3.8 (0.85)	−5.9 (1.52)	<.001**
Total number of loose FI episodes, mean (SE)	10.9 (1.32)	5.9 (1.49)	−5.1 (1.67)	<.001
Total number of FI-free days, mean (SE)	14.1 (0.81)	21.2 (0.88)	7.1 (0.94)	<.001*
Number of controlled bowel evacuations, mean (SE)	23.0 (2.18)	24.4 (2.41)	1.5 (1.81)	.315*
Cleveland Clinic Florida Incontinence Score, mean (SE)	13.4 (0.36)	8.7 (0.50)	−4.7 (0.48)	<.001*
Fecal Incontinence Quality of Life (FIQL) Scores				
Lifestyle, mean (SE)	2.4 (0.10)	2.9 (0.09)	0.5 (0.10)	<.001*
Coping/behavior, mean (SE)	1.7 (0.07)	2.4 (0.11)	0.7 (0.11)	<.001*
Depression/self perception, mean (SE)	2.7 (0.10)	3.2 (0.10)	0.5 (0.11)	<.001*
Embarrassment, mean (SE)	1.8 (0.08)	2.6 (0.11)	0.8 (0.11)	<.001*

SE: standard error.

*One-sample *t*-test; **Wilcoxon onesample test.

**Table 4:** Related AEs with an incidence of >3 events by MedDRA preferred term: safety population.

MedDRA preferred term	Number of events	Maximal intensity			Median time to onset	Median duration	Method of intervention*			Proportion of events resolved
		Mild	Moderate	Severe			None required	Medical treatment	Other	
Adominal pain	3	1	2	0	1.0	8.0	1	2	0	100%
Constipation	5	4	1	0	2.0	3.0	1	4	0	100%
Diarrhea	5	2	3	0	1.0	8.0	3	2	0	100%
Injection site pain	4	2	2	0	1.0	3.0	0	4	0	100%
Perineal pain	3	1	2	0	1.0	6.0	1	2	0	100%
Proctalgia	15	7	7	1	0.0	5.0	9	5	1*	100%
Pyrexia	8	3	4	1	1.0	3.5	2	6	0	100%
Rectal hemorrhage	3	2	1	0	13.0	4.0	2	0	1*	100%
Rectal tenesmus	3	3	0	0	0.0	8.0	3	0	0	100%

MedDRA: medical dictionary of regulatory authorities.

*One event each of proctalgia and rectal hemorrhage were coinciding events with a rectal prolapse that was treated in surgery.

## References

[1] Oliveira L, Wexner SD, Beck DE, Wexner SD (1998). Anal incontinence. *Fundamentals of Anorectal Surgery*.

[2] Kalantar JS, Howell S, Talley NJ (2002). Prevalence of faecal incontinence and associated risk factors: an underdiagnosed problem in the Australian community?. *Medical Journal of Australia*.

[3] Ho YH, Muller R, Veitch C, Rane A, Durrheim D (2005). Faecal incontinence: an unrecognised epidemic in rural North Queensland? Results of a hospital-based outpatient study. *Australian Journal of Rural Health*.

[4] Siproudhis L, Pigot F, Godeberge P, Damon H, Soudan D, Bigard MA (2006). Defecation disorders: a French population survey. *Diseases of the Colon and Rectum*.

[5] Nelson R, Norton N, Cautley E, Furner S (1995). Community-based prevalence of anal incontinence. *Journal of the American Medical Association*.

[6] Perry S, Shaw C, McGrother C (2002). Prevalence of faecal incontinence in adults aged 40 years or more living in the community. *Gut*.

[7] Enck P, Bielefeldt K, Rathmann W, Purrmann J, Tschope D, Erckenbrecht JF (1991). Epidemiology of faecal incontinence in selected patient groups. *International Journal of Colorectal Disease*.

[8] Faltin DL, Sangalli MR, Curtin F, Morabia A, Weil A (2001). Prevalence of anal incontinence and other anorectal symptoms in women. *International Urogynecology Journal and Pelvic Floor Dysfunction*.

[9] Tariq SH, Morley JE, Prather CM (2003). Fecal incontinence in the elderly patient. *American Journal of Medicine*.

[10] Zetterström J, Mellgren A, Jensen LL (1999). Effect of delivery on anal sphincter morphology and function. *Diseases of the Colon and Rectum*.

[11] Abramowitz L, Sobhani I, Ganansia R (2000). Are sphincter defects the cause of anal incontinence after vaginal delivery? Results of a prospective study. *Diseases of the Colon and Rectum*.

[12] Sato T, Konishi F, Minakami H (2001). Pelvic floor disturbance after childbirth: vaginal delivery damages the upper levels of sphincter innervation. *Diseases of the Colon and Rectum*.

[13] Bartlett L, Nowak M, Ho YH (2009). Impact of fecal incontinence on quality of life. *World Journal of Gastroenterology*.

[14] Malouf AJ, Norton CS, Engel AF, Nicholls RJ, Kamm MA (2000). Long-term results of overlapping anterior anal-sphincter repair for obstetric trauma. *The Lancet*.

[15] Wexner SD, Hull T, Edden Y (2010). Infection rates in a large investigational trial of sacral nerve stimulation for fecal incontinence. *Journal of Gastrointestinal Surgery*.

[16] Pinto RA, Sands DR (2009). Surgery and sacral nerve stimulation for constipation and fecal incontinence. *Gastrointestinal Endoscopy Clinics of North America*.

[17] Matzel KE, Lux P, Heuer S, Besendörfer M, Zhang W (2009). Sacral nerve stimulation for faecal incontinence: long-term outcome. *Colorectal Disease*.

[18] Efron JE (2004). The SECCA procedure: a new therapy for treatment of fecal incontinence. *Surgical Technology International*.

[19] Lefebure B, Tuech JJ, Bridoux V (2008). Temperature-controlled radio frequency energy delivery (Secca procedure) for the treatment of fecal incontinence: results of a prospective study. *International Journal of Colorectal Disease*.

[20] Takahashi-Monroy T, Morales M, Garcia-Osogobio S (2008). SECCA procedure for the treatment of fecal incontinence: results of five-year follow-up. *Diseases of the Colon and Rectum*.

[21] Ruppert P, Staimmer D (1998). Fecal incontinence—new surgical treatments. ABS-artificial bowel sphincter. *Krankenpflege Journal*.

[22] Melenhorst J, Koch SM, van Gemert WG, Baeten CG (2008). The artificial bowel sphincter for faecal incontinence: a single centre study. *International Journal of Colorectal Disease*.

[23] Baeten CGMI, Uludag O (2002). Second-line treatment for faecal incontinence. *Scandinavian Journal of Gastroenterology, Supplement*.

[24] Thornton MJ, Kennedy ML, Lubowski DZ, King DW (2004). Long-term follow-up of dynamic graciloplasty for faecal incontinence. *Colorectal Disease*.

[25] Penninckx F (2004). Belgian experience with dynamic gracitoplasty for faecal incontinence. *British Journal of Surgery*.

[26] Rongen MJGM, Uludag O, El Naggar K, Geerdes BP, Konsten J, Baeten CGMI (2003). Long-term follow-up of dynamic graciloplasty for fecal incontinence. *Diseases of the Colon and Rectum*.

[27] Rosen HR (2002). Modern concepts for the treatment of fecal incontinence. *Acta Chirurgica Lugoslavica*.

[28] Rossini CJ, Moriarty KP, Courtney RA, Tashjian DB (2009). Endoscopic treatment with deflux for refluxing duplex systems. *Journal of Laparoendoscopic and Advanced Surgical Techniques*.

[29] Wadie GM, Tirabassi MV, Courtney RA, Moriarty KP (2007). The deflux procedure reduces the incidence of urinary tract infections in patients with vesicoureteral reflux. *Journal of Laparoendoscopic and Advanced Surgical Techniques*.

[30] Kerr LA (2005). Bulking agents in the treatment of stress urinary incontinence: history, outcomes, patient populations, and reimbursement profile. *Reviews in Urology*.

[31] Altomare DF, La Torre F, Rinaldi M, Binda GA, Pescatori M (2008). Carbon-coated microbeads anal injection in outpatient treatment of minor fecal incontinence. *Diseases of the Colon and Rectum*.

[32] Stojkovic SG, Lim M, Burke D, Finan PJ, Sagar PM (2006). Intra-anal collagen injection for the treatment of faecal incontinence. *British Journal of Surgery*.

[33] Tjandra JJ, Lim JF, Hiscock R, Rajendra P (2004). Injectable silicone biomaterial for fecal incontinence caused by internal anal sphincter dysfunction is effective. *Diseases of the Colon and Rectum*.

[34] de la Portilla F, Fernández A, León E (2008). Evaluation of the use of PTQ*™* implants for the treatment of incontinent patients due to internal anal sphincter dysfunction. *Colorectal Disease*.

[35] Kenefick NJ, Vaizey CJ, Malouf AJ, Norton CS, Marshall M, Kamm MA (2002). Injectable silicone biomaterial for faecal incontinence due to internal anal sphincter dysfunction. *Gut*.

[36] Danielson J, Karlbom U, Sonesson AC, Wester T, Graf W (2009). Submucosal injection of stabilized nonanimal hyaluronic acid with dextranomer: a new treatment option for fecal incontinence. *Diseases of the Colon and Rectum*.

[37] Lavelle MT, Conlin MJ, Skoog SJ (2005). Subureteral injection of Deflux for correction of reflux: analysis of factors predicting success. *Urology*.

[38] Decter RM (2001). Vesicoureteral reflux. *Pediatrics in Review*.

[39] Garin EH, Olavarria F, Nieto VG, Valenciano B, Campos A, Young L (2006). Clinical significance of primary vesicoureteral reflux and urinary antibiotic prophylaxis after acute pyelonephritis: a multicenter, randomized, controlled study. *Pediatrics*.

[40] Puri P, Pirker M, Mohanan N, Dawrant M, Dass L, Colhoun E (2006). Subureteral dextranomer/hyaluronic acid injection as first line treatment in the management of high grade vesicoureteral reflux. *Journal of Urology*.

[41] Stenberg A, Lackgren G (1995). A new bioimplant for the endoscopic treatment of vesicoureteral reflux: experimental and short-term clinical results. *Journal of Urology*.

[42] Läckgren G, Wåhlin N, Stenberg A (1999). Endoscopic treatment of children with vesico-ureteric reflux. *Acta Paediatrica, International Journal of Paediatrics, Supplement*.

[43] Läckgren G, Wåhlin N, Sköldenberg E, Stenberg A (2001). Long-term followup of children treated with dextranomer/hyaluronic acid copolymer for vesicoureteral reflux. *Journal of Urology*.

[44] World Medical Association Declaration of Helsinki (1998). Ethical principles for medical research involving human patients. *The Journal Of the American Medical Association*.

[45] World Medical Association Declaration of Helsinki (1996). Ethical principles for medical research involving human patients. *Nursing Ethics*.

[46] World Medical Association Declaration of Helsinki (2002). Ethical principles for medical research involving human patients. *Journal of Postgraduate Medicine*.

[47] World Medical Association Declaration of Helsinki (2004). Ethical principles for medical research involving human patients. *Journal International de Bioéthique*.

[48] Oliveira L, Pfeifer J, Wexner SD (1996). Physiological and clinical outcome of anterior sphincteroplasty. *British Journal of Surgery*.

[49] Rothbarth J, Bemelman WA, Meijerink WJHJ (2001). What is the impact of fecal incontinence on quality of life?. *Diseases of the Colon and Rectum*.

[50] Rockwood TH, Church JM, Fleshman JW (2000). Fecal Incontinence Quality of Life Scale: quality of life instrument for patients with fecal incontinence. *Diseases of the Colon and Rectum*.

[51] Guerra F, Velluti F, Crocetti D, La Torre F (2010). PTQ*™* bulking agent injection for the treatment of fecal incontinence: QoL and manometric evaluation. *Pelviperineology*.

[52] Mellgren A (2010). Fecal incontinence. *Surgical Clinics of North America*.

[53] Levy BF, Tilney HS, Dowson HMP, Rockall TA (2010). A systematic review of postoperative analgesia following laparoscopic colorectal surgery. *Colorectal Disease*.

[54] Hagemeier T, Blau U, Gauruder-Burmester A, Tunn R (2006). Paraurethral abscess developing after mid-urethral Zuidex-injection in women with stress urinary incontinence—management of complications and retrospective comparison with bladder neck located injection technique. *Zentralblatt fur Gynakologie*.

[55] Hilton P (2009). Urethrovaginal fistula associated with 'sterile abscess' formation following periurethral injection of dextranomer/hyaluronic acid co-polymer (Zuidex) for the treatment of stress urinary incontinence—a case report. *British Journal of Obstetrics and Gynaecology*.

[56] Sahai A, Thomas M, Nedas T, Larner T, Niekrash R, Hammadeh MY (2009). Periurethral non-animal stabilized hyaluronic acid/dextranomer injections: efficacy and formation of granuloma/sterile abscess. *Urology*.

[57] Bech L, Sander P, Lose G (2009). Bilateral suprapubic abscessses five years after incontinence surgery. *Ugeskrift for Laeger*.

[58] Zumbé J, Porres D, Degiorgis PL, Wyler S (2008). Obturator and thigh abscess after transobturator tape implantation for stress urinary incontinence. *Urologia Internationalis*.

[59] Leanza V, Garozzo V, Accardi M, Molino A, Conca M, Basile A (2008). A late complication of transobturator tape: abscess and myositis. *Minerva Ginecologica*.

[60] Glavind K, Larsen T (2008). Long-term follow-up of intravaginal slingplasty operation for urinary stress incontinence. *International Urogynecology Journal and Pelvic Floor Dysfunction*.

[61] Sivanesan K, Abdel-Fattah M, Tierney J (2007). Perineal cellulitis and persistent vaginal erosion after transobturator tape (Obtape)—case report and review of the literature. *International Urogynecology Journal and Pelvic Floor Dysfunction*.

[62] Marsh F, Rogerson L (2007). Groin abscess secondary to trans obturator tape erosion: case report and literature review. *Neurourology and Urodynamics*.

[63] Karsenty G, Boman J, Elzayat E, Lemieux MC, Corcos J (2007). Severe soft tissue infection of the thigh after vaginal erosion of transobturator tape for stress urinary incontinence. *International Urogynecology Journal and Pelvic Floor Dysfunction*.

[64] Deffieux X, Donnadieu AC, Mordefroid M, Levante S, Frydman R, Fernandez H (2007). Prepubic and thigh abscess after successive placement of two suburethral slings. *International Urogynecology Journal and Pelvic Floor Dysfunction*.

[65] Benassi G, Marconi L, Accorsi F, Angeloni M, Benassi L (2007). Abscess formation at the ischiorectal fossa 7 months after the application of a synthetic transobturator sling for stress urinary incontinence in a type II diabetic woman. *International Urogynecology Journal and Pelvic Floor Dysfunction*.

[66] Yamada BS, Govier FE, Stefanovic KB, Kobashi KC (2006). High rate of vaginal erosions associated with the mentor ObTape. *Journal of Urology*.

[67] Robert M, Murphy M, Birch C, Swaby C, Ross S (2006). Five cases of tape erosion after transobturator surgery for urinary incontinence. *Obstetrics and Gynecology*.

[68] Rafii A, Jacob D, Deval B (2006). Obturator abscess after transobturator tape for stress urinary incontinence. *Obstetrics and Gynecology*.

[69] Madjar S, Frischer Z, Nieder AM, Waltzer WC (2006). Bladder wall abscess following midurethral sling procedure. *International Urogynecology Journal and Pelvic Floor Dysfunction*.

[70] Goldman HB (2006). Large thigh abscess after placement of synthetic transobturator sling. *International Urogynecology Journal and Pelvic Floor Dysfunction*.

[71] Deval B, Haab F (2006). Management of the complications of the synthetic slings. *Current Opinion in Urology*.

[72] Deval B, Ferchaux J, Berry R (2006). Objective and subjective cure rates after trans-obturator tape (OBTAPE) treatment of female urinary incontinence. *European Urology*.

[73] Babalola EO, Famuyide AO, McGuire LJ, Gebhart JB, Klingele CJ (2006). Vaginal erosion, sinus formation, and ischiorectal abscess following transobturator tape: ObTape implantation. *International Urogynecology Journal and Pelvic Floor Dysfunction*.

[74] Agostini A, De Lapparent T, Bretelle F, Roger V, Cravello L, Blanc B (2006). Abscess of the thigh and psoas muscle after transobturator suburethral sling procedure. *Acta Obstetricia et Gynecologica Scandinavica*.

[75] Tate SB, Franco AV, Fynes MM (2005). Tension-free vaginal tape exposure presenting as a recurrent sterile paraurethral abscess. *International Urogynecology Journal and Pelvic Floor Dysfunction*.

[76] Giles DL, Davila GW (2005). Suprapubic-vaginocutaneous fistula 18 years after a bladder-neck suspension. *Obstetrics and Gynecology*.

[77] Baessler K, Hewson AD, Tunn R, Schuessler B, Maher CF (2005). Severe mesh complications following intravaginal slingplasty. *Obstetrics and Gynecology*.

[78] Kalota SJ (2004). Small intestinal submucosa tension-free sling: postoperative inflammatory reactions and additional data. *Journal of Urology*.

[79] Ghosh T, Banfield PJ, Klazinga DA (2004). An unusual complication of tension-free vaginal tape procedure: recurrent anterior vaginal wall abscess and sinus formation. *Journal of Obstetrics and Gynaecology*.

[80] Game X, Mouzin M, Vaessen C, Malavaud B, Sarramon JP, Rischmann P (2004). Obturator infected hematoma and urethral erosion following transobturator tape implantation. *Journal of Urology*.

[81] Cindolo L, Salzano L, Rot G, Bellini S, D’Afiero A (2004). Tension-free transobturator approach for female stress urinary incontinence. *Minerva Urologica e Nefrologica*.

[82] Sergent F, Sebban A, Verspyck E (2003). Per- and postoperative complications of TVT (tension-free vaginal tape). *Progres en Urologie*.

[83] Romero Maroto J, Prieto Chaparro L, López López C, Quilez Fenoll JM, Bolufer Nadal S (2002). Prolene mesh sling in the treatment of stress urinary incontinence. Integral treatment of pelvic floor anomalies. Long-term results. *Archivos Espanoles de Urologia*.

[84] Kiilholma PJ, Chancellor MB, Makinen J, Hirsch IH, Klemi PJ (1993). Complications of Teflon injection for stress urinary incontinence. *Neurourology and Urodynamics*.

[85] Fahmy MAB, Ezzelarab S (2004). Outcome of submucosal injection of different sclerosing materials for rectal prolapse in children. *Pediatric Surgery International*.

[86] Elmore JM, Scherz HC, Kirsch AJ (2006). Dextranomer/hyaluronic acid for vesicoureteral reflux: success rates after initial treatment failure. *Journal of Urology*.

[87] Q-Med_AB. Deflux Package Insert 2001 http://www.accessdata.fda.gov/cdrh_docs/pdf/P000029c.pdf.

